# Genome-Wide Transcriptome Analysis of Two Contrasting *Brassica rapa* Doubled Haploid Lines under Cold-Stresses Using Br135K Oligomeric Chip

**DOI:** 10.1371/journal.pone.0106069

**Published:** 2014-08-28

**Authors:** Hee-Jeong Jung, Xiangshu Dong, Jong-In Park, Senthil Kumar Thamilarasan, Sang Sook Lee, Yeon-Ki Kim, Yong-Pyo Lim, Ill-Sup Nou, Yoonkang Hur

**Affiliations:** 1 Department of Horticulture, Sunchon National University, Suncheon, Jeonnam, Republic of Korea; 2 Department of Biology, College of Biological Science and Biotechnology, Chungnam National University, Daejeon, Republic of Korea; 3 GreenGene Biotech Inc., Genomics and Genetics Institute, Yongin, Republic of Korea; 4 Department of Horticulture, Chungnam National University, Daejeon, Republic of Korea; Key Laboratory of Horticultural Plant Biology (MOE), China

## Abstract

Genome wide transcription analysis in response to stresses is important to provide a basis of effective engineering strategies to improve stress tolerance in crop plants. We assembled a *Brassica rapa* oligomeric microarray (Br135K microarray) using sequence information from 41,173 unigenes and analyzed the transcription profiles of two contrasting doubled haploid (DH) lines, Chiifu and Kenshin, under cold-treatments. The two DH lines showed great differences in electrolyte leakage below −4°C, but similar patterns from 4°C to −2°C. Cold-treatments induced 885 and 858 genes in Chiifu and Kenshin, respectively. Overall, 134, and 56 genes showed an intrinsic difference in expression in Chiifu and Kenshin, respectively. Among 5,349 genes that showed no hit found (NHF) in public databases, 61 and 24 were specifically expressed in Chiifu and Kenshin, respectively. Many transcription factor genes (TFs) also showed various characteristics of expression. *BrMYB12*, *BrMYBL2*, *BrbHLHs*, *BrbHLH038*, a C2H2, a WRKY, *BrDREB19* and a integrase-type TF were induced in a Chiifu-specific fashion, while a bHLH (Bra001826/AT3G21330), *bHLH*, cycling Dof factor and two Dof type TFs were Kenshin specific. Similar to previous studies, a large number of genes were differently induced or regulated among the two genotypes, but many genes, including NHFs, were specifically or intrinsically expressed with genotype specificity. Expression patterns of known-cold responsive genes in plants resulted in discrepancy to membrane leakage in the two DH lines, indicating that timing of gene expression is more important to conferring freezing tolerance rather than expression levels. Otherwise, the tolerance will be related to the levels of transcripts before cold-treatment or regulated by other mechanisms. Overall, these results indicate common signaling pathways and various transcriptional regulatory mechanisms are working together during cold-treatment of *B. rapa.* Our newly developed Br135K oligomeric microarray will be useful for transcriptome profiling, and will deliver valuable insight into cold stresses in *B. rapa*.

## Introduction

Plants are constantly exposed to a variety of abiotic stresses, including drought, salinity and cold, which has resulted in development of several protective mechanisms against these stresses. Among abiotic stresses, chilling and freezing have strong effects on plant growth and development, which control crop distribution and yield. Plants exposed to low temperatures (LTs) in the range of 0–15°C undergo chilling injury, whereas temperatures below 0°C result in freezing injury. These processes are collectively referred to as cold stress [1]–[3]. Most temperate plants have the ability to obtain cold-tolerance after being exposed to mildly low but nonfreezing temperatures (below 10°C) via cold acclimation [4]. The C-Repeat Binding Factor (CBF) transcriptional pathway plays an important role in cold acclimation and activates the *COR* (COLD RESPONSIVE) genes [5]. In addition to temperature, light acts as an external signal that affects plant growth. Several studies have shown that light is required for cold acclimation, specifically for development of freezing tolerance [6], [7]. Light mediates cold acclimation through a novel low temperature response element (LTRE) referred to as the Z-box element. Genes such as *CBF2, CBF1, CBF3, HY5, PhyB,* and *PhyD* are involved in the light signaling and cold acclimation response pathway. Similarly, the *PIF4* and *PIF7* genes are involved in photoperiodism and the cold acclimation pathway [8], [9].

LT and short photoperiods directly influence the physiological, metabolic and transcriptional factors, resulting in increased freezing tolerance in woody plants such as silver birch [10]. Several studies have reported that freezing tolerance can be increased by overexpressing *CBF* genes in different plant species such as *Brassica napus, Lycopersicon esculentum* [11], wheat and barley [12]. In *Arabidopsis*, short day condition results in up-regulation of the CBF pathway, which increases freezing tolerance, whereas long day condition down-regulates the CBF pathway *via* phytochrome B and two phytochrome-interacting factors, PIF4 and IF7, which decreases tolerance [8]. However, cold-resistance in plants is a very complex trait that involves many different metabolic pathways and cell compartments [4].

Many *Brassica* crop species provide edible roots, leaves, stems, buds, flowers and seeds [13]. Seven groups of vegetable *B. rapa* types have been reported to date, var. *campestris*, var. *pekinensis*, var. *chinensis*, var. *parachinensis*, var. *narinosa*, var. *japonica* and var. *rapa*. Chinese cabbage (*B. rapa* ssp. *pekinen*sis) is one of the most important vegetable crops in Korea, China and other east Asian countries. Two Chinese cabbage lines, Chiffu and Kenshin, have different geographic origins. Specifically, Chiffu is from temperate regions while Kenshin originates from tropical and subtropical regions [14], implying that they will have different responsiveness to temperature stresses. Many oligomeric chips have been developed to identify differentially expressed genes in *B. rapa*, including the Br24K microarray (version 1) [15] and Br300K microarray (version 2) [16]. Lee et al. [16] used the Br24K microarray to examine transcriptomes when the *B. rapa* double haploid line Chiifu was exposed to abiotic stresses. Additionally, expression sequence tags (ESTs) were annotated for *Brassica rapa* based on gene ontologies [17]. Complete elucidation of AP2-ERF transcription factors (TFs) helps identify crop stress mechanisms [18]. However, no genome wide transcriptome analyses have been performed using whole genes or well-designed cold-treatments in *B. rapa*.

Together with the availability of comprehensive sequences of model plants and whole sets of unigenes (http://www.ncbi.nlm.nih.gov), the microarray technique has led to increased understanding of many features of plant biology, including the discovery of important agricultural crop traits. To elucidate the chilling- and freezing-resistance mechanism, we developed version 3 of the microarray (Br135K) using 41,173 unigenes representing whole genes in *B. rapa* (http://www.brassica.info/) [19]. We then used this microarray to comprehensively analyze genes that are up-regulated and down regulated by cold stress.

## Materials and Methods

### Plant materials and cold treatment

Two Chinese cabbage (*Brassica rapa* ssp. *pekinensis*) double haploid (DH) lines, Chiifu and Kenshin, were grown for approximately 4 weeks in a growth chamber at 22°C under a 16 h light/8 h dark photoperiod with a photon flux density of 140 µmol m^−2 ^s^−1^. For cold treatments, plants grown in the growth chamber (C1, K1) were subjected to 4°C for 6 h (C2, K2) followed by 2 h at 0°C (C3, K3), 2 h at −2°C (C4, K4), 2 h at −4°C (C5, K5) and then 4°C for 6 h and 22°C (C6, K6) for 24 h, respectively. C6 and K6 were used to represent plants recovered from cold and freezing stress, respectively.

### Electrolyte leakage test

Immediately after treatment, electrolyte leakage from the cold-stressed and control plants was measured using previously described methods [20], [21], with some modifications. Briefly, 10 leaf discs (1 cm in diameter) were excised from fully expanded leaves of 3–4 plants and placed in a glass tube with 10 ml distilled water. The samples were then incubated on an orbital shaker at 150 rpm for 30 min at room temperature, after which the initial conductivity (I) was measured using CON110 conductivity meter (Oakton Ins. USA). The leaf discs were then kept in a boiling water bath for 10 min, after which they were cooled to room temperature and the final conductivity (F) was measured. The relative electrolyte leakage was calculated using the formula, I/F ×100.

### Construction of Br135K chip

The Br135K microarray (Brapa_V3_microarray, 3′-Tiling microarray) is a high-density DNA array prepared using Maskless Array Synthesizer (MAS) technology by NimbleGen (http://www.nimblegen.com/). Probes are designed from 41,173 genes of *Brassica rapa* accession Chiifu-401-42, a Chinese cabbage [19]. The length of each of the three probes was 60 mers, and probes were designed based on 30 bp that overlapped in 120 bp sequences (60 bp coding sequence plus 60 bp of 3′UTR of each gene), representing 123,647 features. Fifty features were also deposited from five markers (*GUS, GFP, Bar, Kan, Hyg*). Total and polysomal RNA was extracted using the RNeasy Mini kit (Qiagen) and the RNA protect reagent (Qiagen), after which DNA was removed by on-column DNase digestion using an RNase-Free DNase set (Qiagen). Labeling was performed by NimbleGen Systems Inc. according to their standard operating protocol (www.nimblegen.com). The raw data (pair files) were subjected to RMA (Robust Multi-Array Analysis) [21], quantile normalization [22], and background correction as implemented in the NimbleScan software package, version 2.4.27. To assess the reproducibility of the microarray analysis, we repeated the experiment two or three times using independently prepared total RNA. The complete raw microarray data have been deposited in the Omics database of NABIC (http://nabic.rda.go.kr) as enrolled numbers NC-0024-000001-NC-0024-000012.

### Gene chip data analysis

Genes with adj.P.Value or false discovery rate below 0.05 were collected and further selected for those genes with expression greater than or less than atleast one treatment compared with expression at all. Multivariate statistical tests such as clustering, principal component analysis, and multidimensional scaling were performed with Acuity 3.1 (Molecular Devices, U.S.A.). Hierarchical clustering was performed with similarity metrics based on squared Euclidean correlation and average linkage clustering was used to calculate the distance between genes.

### RNA extraction and RT-PCR analysis

Total RNA was extracted from the cold-treated plants using an RNeasy mini kit (Qiagen, USA), after which the RNA was further treated with RNase-free DNase (Promega, USA) to remove genomic DNA contamination. RT-PCR was then performed using an Avian Myeloblastosis Virus (AMV) one step RT-PCR kit (Takara, Japan). The gene specific primers for the stress responsive genes are listed in Table S1 and S2. RT-PCR was performed using 50 ng of cDNA from plants exposed to cold-temperatures. In 0.5 mL PCR tubes, 20 pmol of each primer, 150 µM of each dNTP, 1.2 U of *Taq* polymerase, 1X *Taq* polymerase buffer, and double-distilled water added to a total volume of 20 µL. The PCR cycle consisted of pre-denaturation at 94°C for 5 min followed by 30 cycles of denaturation at 94°C for 30 s, annealing at 58°C for 30 s and extension at 72°C for 45 s, after which the reaction was terminated by an additional extension step for 5 min at 72°C. PCR products were analyzed following through a 1.5% agarose gel.

## Results and Discussion

### Electrolyte leakage of two DH lines

**Figure 1:**
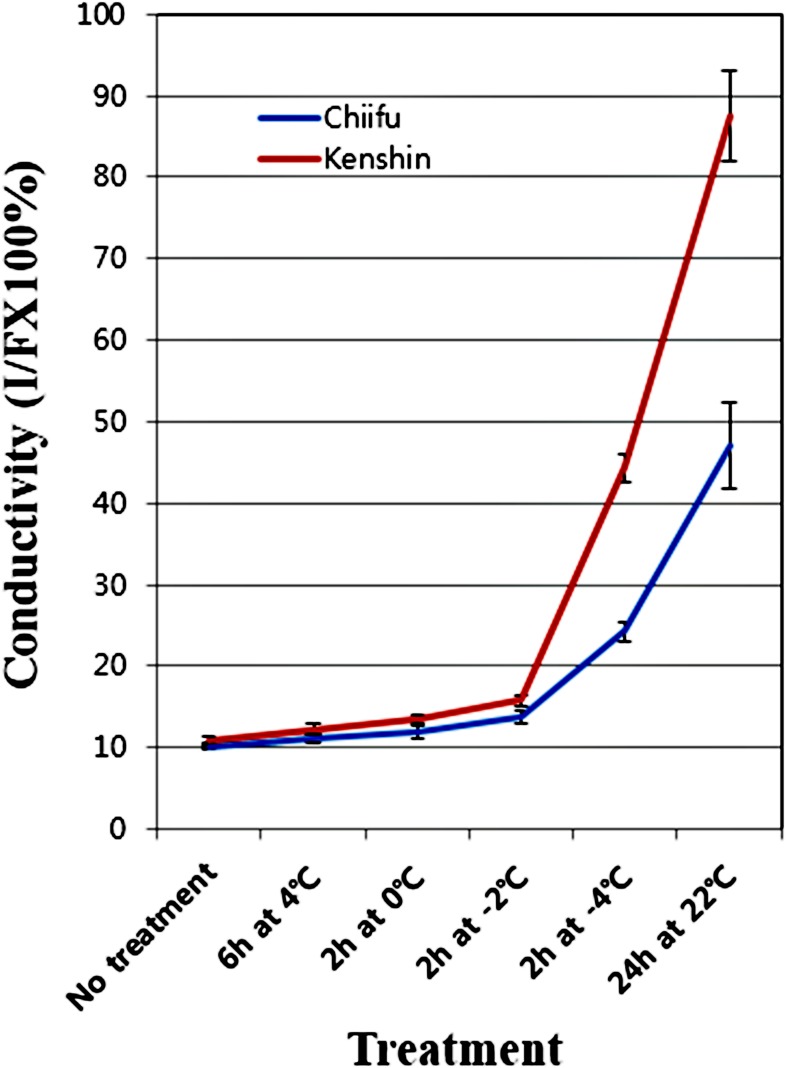
Electrolyte leakage of two contrasting DH lines, Chiifu and Kenshin. The leakage was expressed by % conductivity between initial (I) and final (F) solution.

The two Chinese cabbage DH lines used in this study, Chiifu and Kenshin, have different geographic origins [14], implying the presence of different and line-specific responsiveness to cold-stress. To confirm this property, we checked the electrolyte leakage of leaf samples used in microarray experiments (Figure 1). Since the electrolyte leakage is inversely proportional to freezing tolerance [23], [24], great differences between Chiifu and Kenshin were expected. No great difference was observed in response to cold treatment at −2°C, and 2 fold more leakage was observed in Kenshin than Chiifu at −4°C and in the recovery stage (Figure 1). These findings are similar to those observed upon comparison of cold acclimated and control samples [25]. Similarly, there was no phenotypic damage observed in either line until −2°C, while soggy leaves were observed after treatment at −4°C (more severe damage in Kenshin than Chiifu) (Figure S1). These results imply that the two DH lines may respond equally to chilling stress, but differentially to freezing stress.

### Summary of Br135K microarray

The unigene set of Chiifu and Kenshin was analyzed for similarity/sequence conservation against the TAIR9 database (http://www.arabidopsis.org/) using a BLASTX search. A total of 35,823 (87.0%) unigenes showed significant similarity with *Arabidopsis* genes (Table 1). These results revealed a significant gap in the microarray data shown in Table S3. Specifically, a total of 18,725 *Arabidopsis* genes matched *B. rapa* unigenes, while 5,349 genes (13%) had no *Arabidopsis* counterpart. *B. rapa* usually contains one to five homologous genes corresponding to *Arabidopsis* ortholog (Table S3). *B. rapa* also has 15 disease resistance protein (TIR-NBS-LRR class) genes that are homologous to one *Arabidopsis* gene (AT5G11250). These results support the finding that reduction or duplication of genes in *B. rapa* occurred after triplication. As shown in Table S3, 12,455 genes (30% of the total unigenes) showed PI values of less than 500 in all examined samples, implying that these genes might be expressed in other organs, or induced by other factors.

**Table 1:** General clustering of *B. rapa* unigene sequences.

Descriptive category	Unigene sequence	Sources
Total Unigene sequenced	41,173	BrGP
Probe length	60 mer	
Probes per array	1,23,697	
No. of BLAST hits	35,823	TAIR9 (http://www.arabidopsis.org/)
No blast hits	5,350	

### Overall profiling of gene expression upon cold-treatment

**Figure 2:**
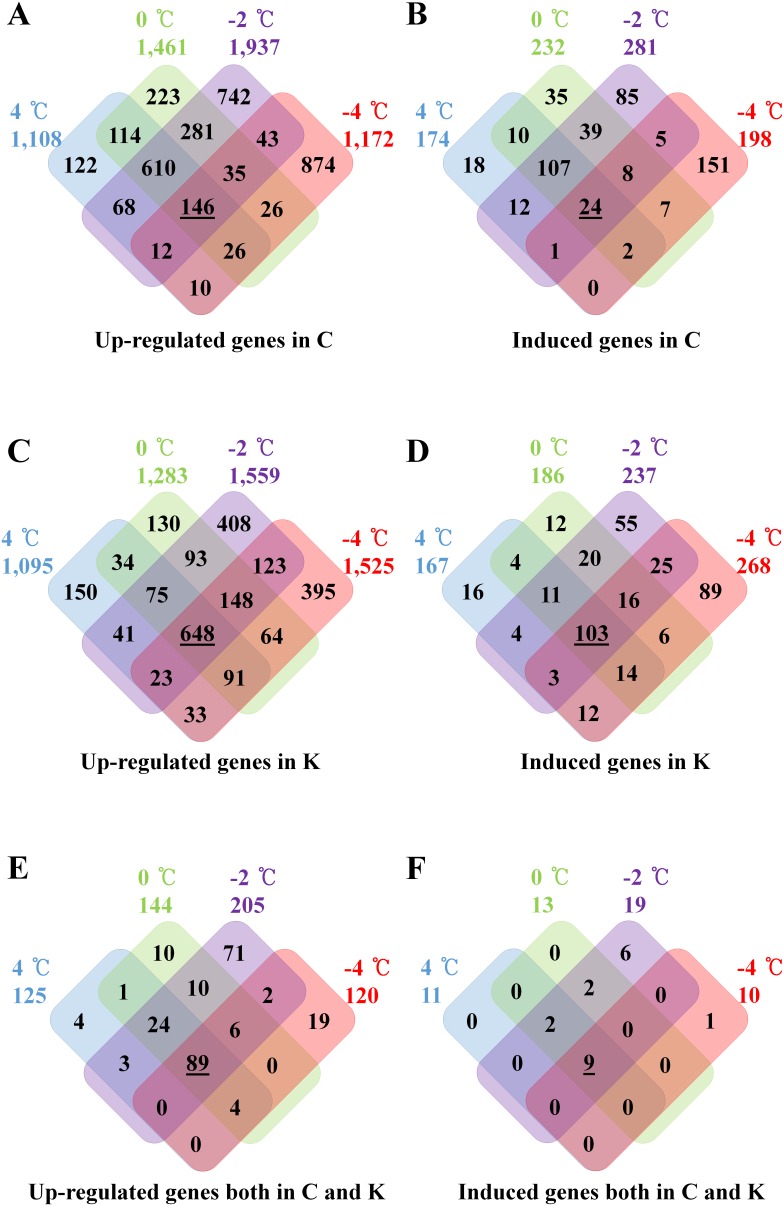
Venn diagram showing up-regulated genes in Chiifu, Kenshin and both upon cold-treatments. C and **K** indicate Chiifu and Kenshin, respectively. **A.** Venn diagram showing 2-folds up-regulated genes in Chiifu by LT treatment. Underlined number indicates that genes were continually up-regulated from 4°C to −4°C treatments. **B.** Venn diagram showing induced genes in Chiifu by LT treatment. Underlined number indicates that genes were continually induced from 4°C to −4°C treatments. **C.** Venn diagram showing 2-folds up-regulated genes in Kenshin by LT treatment. Underlined number indicates that genes were continually up-regulated from 4°C to −4°C treatments. **D.** Venn diagram showing induced genes in Kenshin by LT treatment. Underlined number indicates that genes were continually induced from 4°C to −4°C treatments. **E.** Venn diagram showing 2-folds up-regulated genes in both Chiifu Kenshin by LT treatment. Underlined number indicates that genes were continually up-regulated from 4°C to −4°C treatments. **F.** Venn diagram showing induced genes in both Chiifu Kenshin by cold treatment. Underlined number indicates that genes were continually induced from 4°C to −4°C treatments.

**Figure 3:**
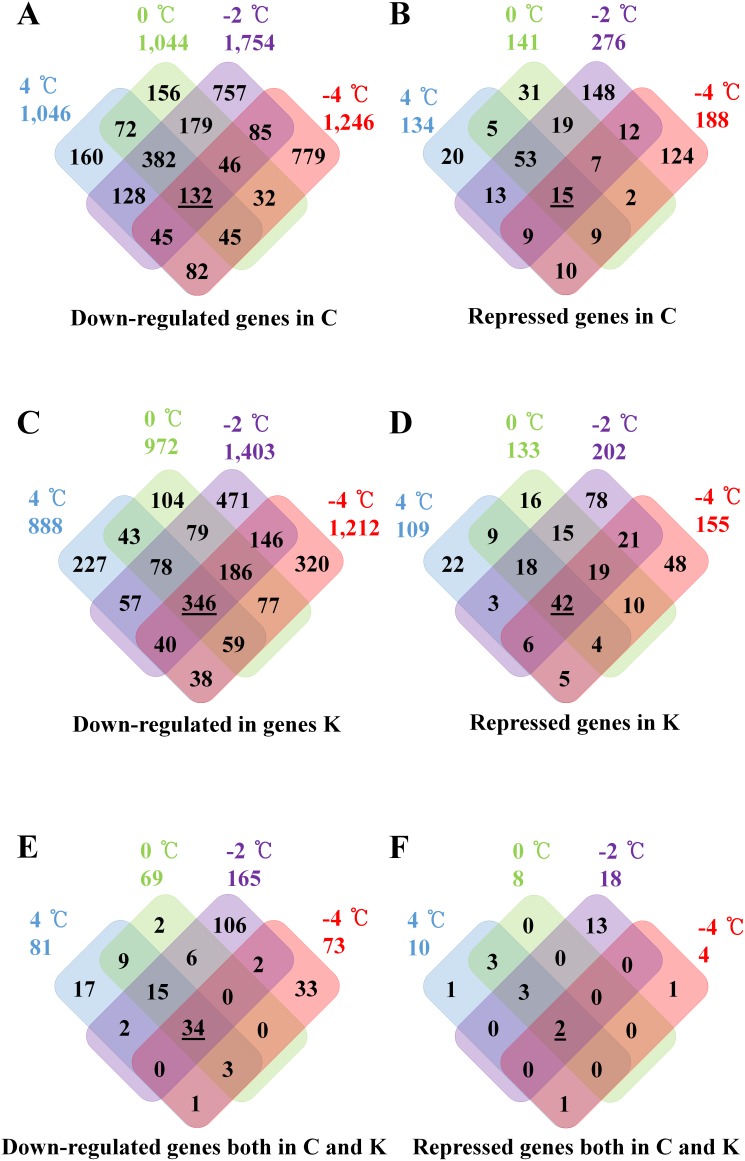
Venn diagram showing down-regulated genes in Chiifu, Kenshin and both upon cold-treatments. C and **K** indicate Chiifu and Kenshin, respectively. **A.** Venn diagram showing 2-folds down-regulated genes in Chiifu by LT treatment. Underlined number indicates that genes were continually down-regulated from 4°C to −4°C treatments. **B.** Venn diagram showing repressed-genes in Chiifu by LT treatment. Underlined number indicates genes were continually repressed from 4°C to −4°C treatments. Repressed genes indicate that genes whose PI value should be above 1000, but PI values of the comparative treatment below 500. **C.** Venn diagram showing 2-folds down-regulated genes in Kenshin by LT treatment. Underlined number indicates that genes were continually down-regulated from 4°C to −4°C treatments. **D.** Venn diagram showing repressed genes in Kenshin by LT treatment. Underlined number indicates that genes were continually repressed from 4°C to −4°C treatments. **E.** Venn diagram showing 2-folds down-regulated genes in both Chiifu and Kenshin by LT treatment. Underlined number indicates that genes were down-regulated from 4°C to −4°C treatments. **F.** Venn diagram showing repressed genes in both Chiifu and Kenshin by LT treatment. Underlined number indicates that genes were continually repressed from 4°C to −4°C treatments.

Chiifu and Kenshin that had been grown for 4-weeks were subjected to various chilling and freezing temperatures and expression profiles were analyzed using various methods (Figure 2 and 3). In the microarray data, 12,455 genes (30%) showed PI values of less than 500 among all samples tested and were therefore eliminated from subsequent expression pattern analysis. A large number of genes were up-regulated in Chiifu by cold-treatments. Specifically, 1,108 genes were up-regulated at 4°C, 1,461 at 0°C, 1,937 at −2°C and 1,172 at −4°C (Figure 2A; Table S4A). These numbers overlapped among cold-treated samples, and most genes were also up-regulated in Kenshin. Many genes up-regulated by 4°C treatment were highly expressed in Chiifu until treatment at −4°C treatment, including *BrCOL2* (Bra021464, Bra001043) and *BrCOR15B* (Bra000263, Bra000265). Conversely, *BrCOL1* (Bra023541), *BrCBF1* (Bra010463), *BrCBF3* (Bra010461), *BrLHY* (Bra030496, Bra033291) and *BrCCA1* (Bra004503) were still expressed following −2°C treatment. These findings indicate that most genes associated with cold stress (4°C) treatments are expressed at the same levels following exposure to freezing temperatures. Overall, 146 genes were up-regulated in all cold-treated samples, most of which were also up-regulated in Kenshin. Up-regulated and down regulated genes were further subdivided into induced and repressed, respectively, at once so called unique genes: 885 genes belong to this category (Figure 2B; Table S4B). Among these, the expression of 24 genes was expressed in all cold-treatments. *BrCOR15B* (Bra000263) and *BrSTH* (salt tolerance homologue, Bra021734) were greatly up-regulated, but they were also up-regulated in Kenshin. Only *BrPCR2* (PLANT CADMIUM RESISTANCE 2, Bra026181) expression was Chiifu-specific, implying that it may control cold-signaling in relation to freezing tolerance because *B. juncea* PCR1 is highly homologous to BrPCR2, which regulates calcium transport [26].

A large number of genes were also up-regulated in Kenshin by cold-treatments: 1,095 at 4°C, 1,283 at 0°C, 1,559 at −2°C and 1,525 at −4°C (Figure 2C; Table S4C). Overall, 648 genes were up-regulated in all cold-treated samples. Expression patterns of these genes were similar to those in Chiifu; however, the number of genes that were up-regulated in Kenshin was larger than that of Chiifu. Among up-regulated genes in Kenshin, the expression of 858 genes was induced and the expression of 103 genes was high in all cold-treated samples (Figure 2D; Table S4D). Bra014525 (AT3G59810, small nuclear ribonucleoprotein family protein) and Bra004139 (AT5G38890, nucleic acid-binding, OB-fold-like protein) expression was Kenshin-specific.

A total of 243 genes were up-regulated by cold-treatments in both genotypes (Figure 2E; Table S4E), implying that these are genes capable of common responsiveness. Among them, Bra000263 (*BrCOR15B*), Bra001043 and Bra021464 (*BrCOL2*, CONSTANS-like 2), and Bra001449 (unknown protein) were very highly up-regulated, but not significantly different between genotypes. Bra001449 (unknown protein) would be particularly important for further research of freezing tolerance. Bra022770 (one of *CBF1*) was up-regulated over 2-fold by treatment at−4°C, indicating that two alleles for one gene may result in differential regulation in response to different low temperature treatments. Among the up-regulated genes, 20 were induced in both genotypes with similar patterns by cold-treatments (Figure 2F; Table S4F), which implies their important roles in cold-response.

Differential regulation of gene expression includes both up- and down-regulation, which may be important for determination of phenotypes such as freezing tolerance. Down-regulated genes may indicate repressed or inhibited expression in response to cold-treatments. Numerous genes were down-regulated in Chiifu: 1,046 at 4°C, 1,044 at 0°C, 1,754 at −2°C and 1,246 at −4°C (Figure 3A; Table S5A). Again, these genes overlapped among cold-treated samples. A total of 132 genes were commonly down-regulated in all cold-treated samples, most of which have functions that have not yet been identified. Among down-regulated genes, 15 were greatly repressed in Chiifu from all cold-treated samples, but only two unknown genes (Bra000755 and Bra039700) showed Chiifu-specific repression (Figure 3B; Table S5B). Conversely, Kenshin-specific gene decreases or repression are expected to be associated with defect of the freezing tolerance. Numerous genes were also down-regulated in Kenshin: 888 at 4°C, 972 at 0°C, 1,403 at −2°C and 1,212 at −4°C (Figure 3C; Table S5C). Among these, 599 genes were repressed in Kenshin, but 42 genes, including Bra013044, Bra024521 and Bra034636, were repressed in all cold-treated samples (Figure 3D; Table S5D).

Genes down-regulated in both genotypes in response to cold treatment may indicate common factors that show decreases in expression owing to cold. Among the 388 commonly down-regulated genes, 34 were down-regulated in both genotypes by cold-treatment, including several TFs (*BrMYB51* (Bra016553), *BrMYB305* (Bra001941), *BrDREB26* (Bra016400), *BrERF6* (Bra021049, Bra040158) and *BrERF104* (Bra012938)) (Figure 3E; Table S5E). Two of the 388 down-regulated genes, Bra010210 (Integrase-type DNA-binding superfamily protein) and Bra016462 (AT1G20520, unknown protein), were repressed in both genotypes (Figure 3F; Table S5F).

### Intrinsic transcriptome differences between Chiifu and Kenshin prior to cold-treatments

Based on the membrane leakage test (Figure 1), Chiifu and Kenshin may undergo different regulation of the CBF regulon or other pathways, and the two DH lines may have intrinsically different sets of genes to resist freezing temperature. To confirm this, we analyzed the microarray data in various ways.

Different phenotypes resulting from variations in gene expression have been observed in different ecotypes of rice and *Arabidopsis* [27], [28]. In addition, previous studies have suggested that highly constitutive gene expression prior to abiotic stress treatment might confer constitutive stress tolerance to tolerant genotypes in several plants, including salt tolerance in *Arabidopsis* [29] and rice [30], heat-stress tolerance in tomato [31], and chilling tolerance in rice [27]. Accordingly, intrinsic differences in certain sets of genes determine the capability for resistance to abiotic and biotic stresses. To explore the intrinsic differences in gene expression, we analyzed gene expression levels in Chiifu and Kenshin under normal growth conditions as well as cold-treatments. Specifically, we selected genes specifically expressed in either DH line in all samples. Genotype-specific genes were defined as those that had PI values greater than 1,000 in all samples in one genotype, but less than 500 in all samples of another genotype. Overall, 134 and 56 genes were specific to Chiifu and Kenshin, respectively (Table 2; Table S6). Based on information describing *Arabidopsis* homologies, these genes were grouped into different biological processes, including response to stress, transport process, etc. The largest group comprised genes involved in response to stress processes (29 genes for Chiifu and 3 genes for Kenshin). Chiifu-specific genes included several disease resistant protein (TIR-NBS-LRR class) genes, as well as *BrWRKY20* and BrWRKY33, while Kenshin genes included *BrCYP81D1, BrRLP6* (receptor like protein 6) and *BrICE2*. Many genes that have no known-counterpart (no hit found, NHF) in the public database showed Chiifu- or Kenshin-specific expression, implying that some might be related to the intrinsic tolerance to freezing stress.

**Table 2:** Functional categorization of genotype-specific expressed genes.

Functional groups	Chiifu- specific	Kenshin-specific
Chloroplast/mitochondrion	8	2
Developmental processes	8	2
Hydrolase activity	4	3
Ion binding	0	1
Oxidoreductase activity	1	1
Protein phosphorylation	3	6
Protein ubiquitination	1	2
Response to stress	29	3
Transport process	5	2
Other	9	2
Unknown biological processes	18	14
NHF	48	18
Total	134	56

Functional classifications were carried out using *Arabidopsis* GO annotation tool (http://www.arabidopsis.org/tools/bulk/go/index.jsp) based on the *Arabidopsis* homologues information. NHF, no_hit_found.

### Identification of cold responsive genes

The membrane leakage test of Chiifu and Kenshin (Figure 1) may imply that cold responsive or freezing tolerant genes have to be expressed differentially at −4°C. Otherwise; their expression must start at least at −2°C and continue to −4°C. To analyze this relationship, we examined transcriptome profiles with respect to cold-treatments and induction of gene expression.

**Figure 4:**
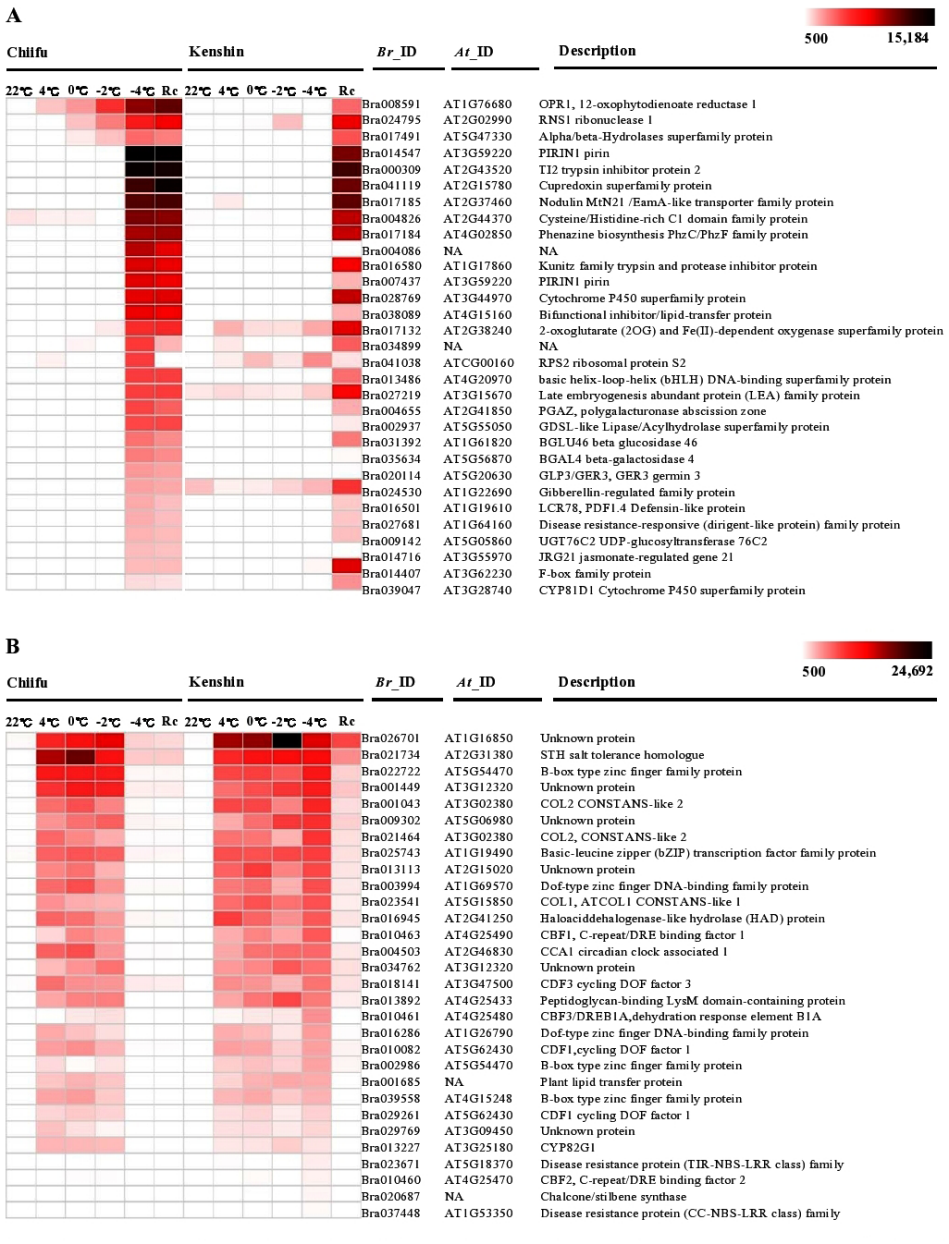
Transcriptome analyses of Chiifu and Kenshin shown by heat maps. Heat maps of expression profiles of top-30 ranked induced genes upon −4°C treatment in Chiifu (A) and Kenshin (B) were compared. Absolute expression values are scaled by PI values. Detailed transcriptome information was described in Table S9 for Chiifu and Table S10 for Kenshin.

Overall, 200 and 271 genes in Chiifu and Kenshin were induced in response to treatment at −4°C (which also include many genes induced by other cold-treatments), respectively (Table S7 and S8). The heat map of expression profiles of the top-30 ranked genes is shown in Figure 4. We found a very peculiar pattern of expression and genotype-specificity. Except for three genes, *BrOPR1* (12-oxophytodienoate reductase), *BrRNS1* (ribonuclease 1) and alpha/beta-hydrolases, all genes differentially expressed by Chiifu were specifically induced by −4°C treatment and continued their expression until the recovery stage (Figure 4A). However, these genes were only expressed in the recovery stage in Kenshin. This difference may be associated with freezing tolerance difference in two genotypes, but there is currently no experimental data supporting this assumption. Pirin1 (*BrPRN1*, AT3G59220)(Bra014547) is one example of this category. Pirin1 is an effector response molecule mediating blue light and ABA signaling that plays a role in ABA response and activates *Lhcb* expression [32]. In contrast to Chiifu, the top-30 ranked genes induced in Kenshin at −4°C were rarely expressed in Chiifu and were largely involved in abiotic stresses, including cold (Figure 4B). These genes included Bra021734 (*BrSTH*, salt tolerance homologue), *BrLHY1*, *BrCCA1*, and *BrCBF1*, *2* and *3*. In several plants, the CBF pathway and circadian clocks are known to be associated with cold acclimation and freezing tolerance [8], [9]. Unexpectedly, these genes were expressed at freezing temperature in Kenshin rather than Chiifu in our study. This discrepancy may have occurred because 1) transcripts levels did not reflect protein levels owing to protein degradation and/or other mechanisms that might be important in *B. rapa* freezing tolerance, 2) different sets of genes might participate in freezing tolerance in a species-specific manner, 3) expression of intrinsic genes might be more important to freezing tolerance in Chinese cabbage.

The membrane leakage test revealed that freezing-responsive genes might also start to be expressed after −2°C treatment. To confirm this, 281 genes induced in Chiifu and 178 induced in Kenshin by −2°C treatment (which also include many genes induced by other cold-treatments) were analyzed. Again, most genes belonging to Chiifu were also induced in Kenshin, until the next treatment, at −4°C, in Kenshin. Most genes for this category were induced by chilling treatment (4°C), and their expression levels were maintained at −2°C for Chiifu and −4°C for Kenshin (Figure S2). These genes included known CBF pathway-related genes, such as *BrCBF1, BrCBF3, BrCOR15B*, and *BrCCA1*. However, the patterns were similar to those observed at −4°C. These results also indicate that timing of gene expression is more important to conferring freezing tolerance than expression levels. In Kenshin, increases in the transcript levels were not associated with freezing tolerance; rather, it appears to be a hypersensitive response of freezing-associated genes, similar to climacteric respiration. Particularly, Chiifu-specific genes induced by −2°C treatment, whose functions have not been confirmed in cold-response, are likely related to Chiifu’s freezing tolerance. Therefore, we analyzed genes that were up-regulated by over 4-fold and induced upon −2°C treatment (Figure S3, Table S11). Most genes in this category were associated with ion transport, including that of cadmium, indicating possible involvement of the ion channel in freezing tolerance signaling. In plants, cold stimulus is sensed by histidine kinases, receptor kinases, calcium sensors, ion channels and phospholipases [2], [4]. However, with the exception of ion channels, most genes related to cold sensors showed similar expression between Chiifu and Kenshin, supporting the importance of ion transport in Chinese cabbage.

Most genes induced by freezing temperatures (−2°C and −4°C) were first expressed at 4°C and their levels remained steady until −4°C or the recovery stage. Therefore, we analyzed 174 Chiifu- and 167 Kenshin-specific genes induced by 4°C treatment (Table S12). As shown in Figure S4, most of the top-30 ranked genes were expressed in all treated samples in both genotypes, indicating their importance in the common freezing tolerance response or cold-acclimation. Among these, we selected genes showing genotype-specific expression in response to cold-treatments (Table 3; Table S13). Most genes were involved had unknown function, indicating their possible roles in freezing tolerance.

**Table 3:** Genotype-specific induced genes by cold-treatments.

Br_ID	At_ID	Description	Fold Change									
			Chiifu					Kenshin				
			4°C/22°C	0°C/22°C	−2°C/22°C	−4°C/22°C	Rc/22°C	4°C/22°C	0°C/22°C	−2°C/22°C	−4°C/22°C	Rc/22°C
Bra009716	AT5G23940	PEL3, PERMEABLE LEAVES3 protein	41.3	26.4	41.1	14.9	16.1	0.6	0.8	0.8	1.2	0.8
Bra021165	AT3G16160	Tesmin/TSO1-like CXC domain-containing protein	17.5	13.4	17.6	2.1	3.3	1.1	0.6	2.1	1.3	0.8
Bra026181	AT1G14870	PCR2 PLANT CADMIUM RESISTANCE 2	7.7	19.4	10.4	7.1	7.2	0.9	1.0	0.7	0.4	1.5
Bra007003	AT3G53470	Unknown protein	7.0	9.5	7.6	4.7	4.3	0.8	1.0	1.1	0.8	1.9
Bra036465	AT2G20340	AAS, aromatic aldehyde synthase	11.1	15.7	16.4	1.0	1.1	0.4	0.4	0.6	0.4	0.3
Bra033985	AT1G72260	THI2.1.1 thionin 2.1	11.0	12.2	7.4	1.8	1.5	2.0	1.4	1.5	1.2	1.5
Bra017371	AT2G03760	SOT12 sulphotransferase 12	9.6	6.6	5.9	1.1	0.9	0.6	1.1	0.8	0.7	0.3
Bra017368	AT2G03760	SOT12 sulphotransferase 12	4.7	3.3	3.1	0.8	0.8	0.3	0.5	1.6	0.3	0.5
Bra017369	AT2G03760	SOT12 sulphotransferase 12	3.1	3.5	2.4	0.7	1.5	1.1	1.1	1.7	1.6	0.6
Bra035271	AT5G49690	UDP-Glycosyltransferase superfamily protein	7.4	5.1	5.6	1.0	1.7	1.1	0.7	0.9	0.8	0.9
Bra034094	AT3G10120	Unknown protein	4.9	4.0	3.4	1.1	1.2	1.6	0.8	1.0	2.2	1.6
Bra039899	AT5G54450	Protein of unknown function (DUF295)	4.2	3.9	3.1	0.9	1.2	1.5	1.1	1.3	1.1	0.9
Bra024091	AT4G30230	Unknown protein	4.2	4.2	2.0	1.1	1.0	1.6	1.3	1.9	1.0	0.9
Bra009220	AT5G06720	ATPA2, PA2 peroxidase 2	3.8	7.0	9.1	0.9	0.7	0.6	0.4	1.4	0.5	0.5
Bra014129	NA	NA	3.6	3.8	3.4	1.2	1.1	0.9	1.9	1.0	0.6	0.8
Bra021122	AT3G15620	UVR3 DNA photolyase family protein	3.1	3.1	3.8	1.3	1.2	1.4	1.0	1.8	1.3	1.1
Bra014525	AT3G59810	Small nuclear ribonucleoprotein family protein	1.1	0.6	0.5	0.6	0.5	8.3	8.6	6.8	8.9	1.6
Bra001343	AT3G08810	Galactose oxidase/kelch repeat superfamily protein3	0.9	1.5	1.1	0.2	0.4	7.1	7.6	4.7	10.9	1.6
Bra020662	NA	NA	0.9	2.1	1.0	1.3	1.4	6.1	5.2	4.5	4.1	2.3
Bra024410	AT5G65690	PCK2, PEPCK phosphoenolpyruvate carboxykinase 2	0.9	1.4	1.9	2.8	2.6	4.8	4.2	2.0	6.3	8.3
Bra018160	AT3G47340	Glutamine-dependent asparagine synthase 1	0.8	1.2	0.5	1.0	0.9	4.5	4.5	2.2	5.0	4.2
Bra030039	AT1G27330	Ribosome associated membrane protein RAMP4	1.1	1.2	1.6	1.3	1.1	4.0	3.7	4.0	1.4	2.6
Bra012272	AT1G21210	WAK4 wall associated kinase 4	1.1	1.0	1.5	0.6	0.7	3.7	3.7	3.5	1.9	0.4

In addition, our microarray deposited 5,349 genes that have NHF in public databases yet. Among them, 61 and 24 genes were expressed in Chiifu and Kenshin, respectively, 8 genes were induced in both genotypes, and some were differentially expressed (Table S14).

**Figure 5:**
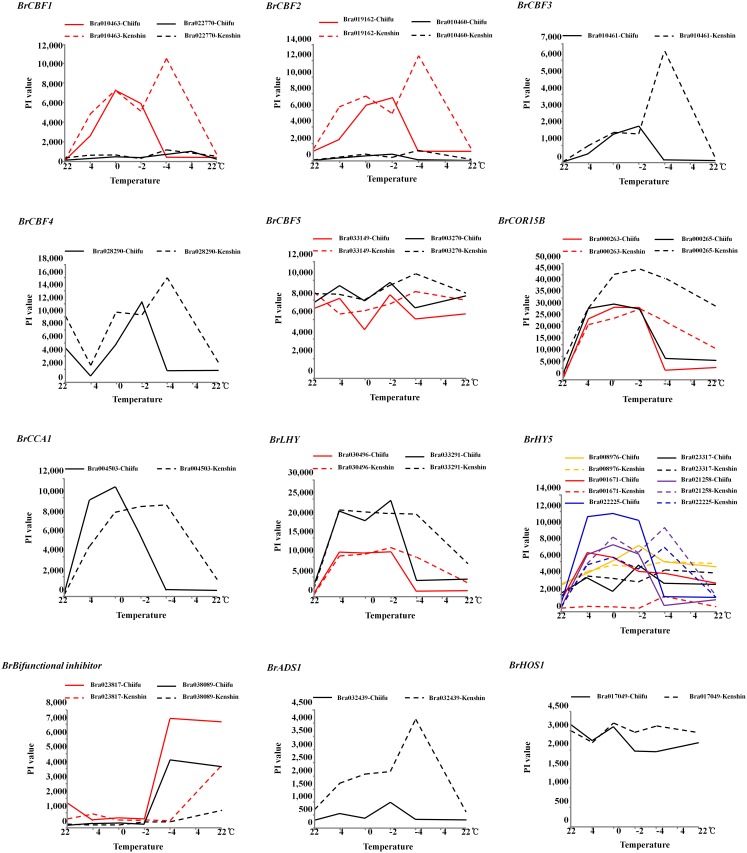
Expression of genes involved in freezing tolerance and genotype specific-expressed genes. PI values were taken from microarray data (Table S1).

The Chinese cabbage microarray showed unexpected results; specifically, the expression of known freezing tolerance-related genes in Kenshin was more sustainable or higher than that in Chiifu. To clarify these findings, we selected several core freezing tolerance-related genes and genotype specific-genes (Figure 5). With the exception of *BrCBF5* and *BrHOS1,* the expression of *BrCBFs, BrCOR15B, BrCCA1* and *BrHY5* was more pronounced in Kenshin, particularly at −4°C). Chiifu-specific ‘Bifunctional inhibitor’ and Kenshin-specific ‘*BrADS1*’ showed high differences in expression between the two genotypes. Overexpression of the *Arabidopsis ADS1* gene, which encodes a plant homologue of the mammalian and yeast acyl-CoA delta9 desaturase, decreased the level of total saturated fatty acids in seeds, but lipid composition was not predictable, suggesting a complex mechanism is involved in the regulation of fatty acid metabolism [33]. Again, our results suggest that timing of gene expression is more important to conferring freezing tolerance than expression levels, accordingly the expression levels in Kenshin appear to be hypersensitive responses.

Chilling injury causes structural and physiological defects [2], reduction in photosynthesis [34] and oxidative stress [35]. Conversely, freezing injury causes leaking cellular membrane and severe cellular dehydration as a result of ice formation in intercellular space [2]. Freezing tolerance is associated with lipid membrane remodeling and composition, but expression of lipid metabolism-related genes and *Sensitive to Freezing 2* (*SFR2*) did not differ between Chiifu and Kenshin.

Cold acclimation confers survival of plants at freezing temperature via pre-exposure to low, nonlethal temperature (below 10°C) [36], [6]. This process involves transcriptional and metabolic changes [37], [4]. During cold-acclimation, LEA (late embryogenesis abundant) and COR protein [35] and solutes [38] accumulate. Plasma membrane H^+^-ATPase activity and fatty acid composition, aquaporins, clathrins, and dynamin-related proteins change significantly during this process [39], [25]. In addition, expression of antioxidant genes [40], TIR-NBS genes [41], photosynthesis-related genes [34], and a lipid desaturase gene [36] is closely related to freezing tolerance in plants. However, most genes related to freezing tolerance described above showed almost no difference between Chiifu and Kenshin, except for two genes (Bra018649, Bra018649) corresponding to one *Arabidopsis* gene AT1G01860 (LEA hydroxyproline-rich glycoprotein family protein gene). These results indicate that freezing tolerance in Chinese cabbage might occur via a different mechanism than in other plants.

Transfer of *Arabidopsis* plants to low temperature induced expression of *CBF1, −2* and −*3*, which was followed about 3 h later by induction of CBF-targeted *COR* genes. Constitutive over-expression of *CBF1, −2*, or *–3* at warm temperatures increased freezing tolerance by leading to constitutive expression of *COR* genes [6]. This scenario is closely related to circadian rhythm [8], [9]. However, we found similar patterns of expression in all of these genes, with no clear difference between Chiifu and Kenshin.

ICE1 (Inducer of CBF expression 1) is a TF gene that induces the expression of *CBFs*. This gene has shown constitutive expression in all *Arabidopsis* tissues, and its over-expression confers freezing tolerance [42]. ICE1 levels are also correlated to *COR* gene expression and regulated by protein levels (or protein degradation) [43]. The up-stream component of ICE1, *Arabidopsis HOS1* (high expression of osmotically responsive gene 1), is an ubiquitin E3 ligase that exerts a negative control against cold response and degrades ICE1 [43]. Expression of transcription factors as well as CBF regulon genes differed among various *Arabidopsis* ecotypes upon cold stress, and significant non-synonymous amino acid changes were observed in the coding regions of the CBF regulon genes [42], indicating possible involvement of protein stability. Our microarray analysis showed that there was no difference in expression of the aforementioned genes between Chiifu and Kenshin, indicating possible regulation at protein levels.

### Expression analysis of transcription factors (TFs)

Transcription factors control a number of genes that participate in the determination of a specific trait, so their effects will be more potent than those of structural genes. We analyzed TFs deposited on a 135 K microarray. About 1,855 TFs genes were composed of genes for MYB (305), bHLH (233), NAC (191), C2H2 zinc binding (159), WRKY (150), bZIP (108), GATA (74), Dof (63), MADS (62), TCP (41), HS (39), GRAS (34), DREB (25), PLATZ (23), and others (348) (Table S15). Among these, 581 TFs were constitutively expressed with values of over 1000 PI in all samples. Particularly, *BrGATA2* (Bra004886, AT2G45050), *BrbZIP53* (Bra007679, AT3G62420), *BrRGA1* (GRAS family transcription factor family protein; Bra017443, AT2G01570), *BrNAC069* (Bra036327, AT4G01550), *BrMYB91* (ATPHAN, AS1; Bra005177, AT2G37630), zinc finger (C2H2 type) family protein (Bra008621/Bra006357, AT5G16470), and others (*BrABI4*; Bra000178, AT2G40220: *BrSPT16* global transcription factor C; Bra000719, AT4G10710) were constitutively and very highly (PI values >20,000) expressed in all samples of both genotypes. These genes play a distinct and crucial role in *Arabidopsis*; namely, regulation of photomorphogenesis by *GATA2* through antagonistic regulation of gene expression and protein degradation by integrating brassinosteroid and light signals [44], regulation of seed maturation by *bZIP53* [45], particular roles during seed germination by *RGA1* [46], regulation of leaf morphology by maintaining the DNA methylation level by *MYB91* [47], regulation of transcription of stress-responsive genes by *ABI4* [48], and assistance with transcription progress as a part of the chromatin remodeling complex FACT by *SPT16* [49]. The very high levels of expression in all tested samples in our microarray implies that many *B. rapa* TFs might have different roles from their *Arabidopsis* counterparts, even though they show high identity in protein sequences.

There are five genes that show genotype-specific expression, regardless of cold-treatments. Three Chiifu-specific expressed TFs are *PLATZ* transcription factor family proteins (Bra023280), MADS-box transcription factor family protein (Bra035685) and *BrWRKY33* (Bra000064), while two Kenshin-specific TFs are *MYB*-like transcription factor family protein (Bra012471) and a bHLH (*BrICE2*, Bra019794). We should pay particular attention to the TFs BrWRKY33 and BrICE2, because, in *Arabidopsis*, WRKY33 is a heat-tolerant responder [50] and ICE2 is a freezing tolerant protein [51]. This also indicates the different regulation of freezing tolerance among plants.

Several TFs were induced by cold-treatments in both Chiifu and Kenshin, which implies their association with cold-responsiveness directly or indirectly. These include two DREBs (*BrCBF1*, Bra010463; *BrCBF2*, Bra010461), three bHLHs (Bra009022; Bra013889; *BrKDR*, Bra016299), four Dof TFs (*BrCDF1*, cycling Dof factor 1, Bra010082/Bra029261; *BrCDF2*, Bra028437; Bra003994; Bra016286), *BrNAC005* (Bra033303) and a bZIP (Bra025743).

Some TFs also showed either Chiifu-specific or Kenshin-specific genotype specificity in their expression upon cold-treatment. TFs induced in a Chiifu-specific fashion included *BrMYB12* (Bra000453), *BrMYBL2* (Bra007957), bHLHs (Bra013486 and Bra022753), *BrbHLH038* (Bra014658), a C2H2 (Bra004300, Bra033944), a WRKY (Bra020196, Bra020197), *DREB19* (Bra005113) and an integrase-type TF (Bra029302), which were expressed at −4°C and continued to be expressed to the recovery stage (Rc) in Chiifu, but were only expressed during the Rc in Kenshin. Only one TF, *BrABR1* (Bra037794), started to be expressed at 0°C and continued to Rc in Chiifu, but was only expressed at Rc in Kenshin. Among these TFs, *BrMYB12, BrMYBL2, BrWRKY38, BrDREB19* and *BrABR1* might be associated with defenses and stresses based on previous studies [52]–[54]. Specifically, these TFs might be important in freezing tolerance in Chiifu. Conversely, several genes were specifically expressed in Kenshin, including a bHLH (Bra001826/AT3G21330), a bHLH (Bra014672/AT3G56770), cycling Dof factor (Bra018141) and two Dof type TFs (Bra003994 and Bra024683).

In addition to TFs showing up-regulated expression upon cold-treatment, expression of many genes was differently regulated, being increased or decreased in both genotypes or increased or decreased only in Chiifu. These genes might also play an important role in freezing tolerance. The following genes were commonly increased in both genotypes upon cold-treatment: *BrMYB7* (Bra013055), *BrMYB59* (Bra002533), *bHLH* (Bra000291, Bra004748), *BrNAC087* (Bra002148), a bZIP (Bra021735), *BrHSFA2* (Bra000557), *BrHSFB2A* (Bra029292), *BrCBF2* (Bra019162), *BrDEAR2* (Bra012140), *BrDREB2B* (Bra029889), *BrEDF3* (ethylene response DNA binding factor 3, Bra026509) and *BrDDF1* (Integrase-type DNA-binding superfamily protein, Bra026963). Heat shock transcription factor A2 (HsfA2) acts as a key component of the Hsf signaling network involved in cellular responses to various types of environmental stress [55]. However, our data indicate that HSFA2 may play a role in cold-response. Conversely, *NAC084* (Bra008788) expression was only increased at −4°C and Rc in Chiifu. The expression of the following TFs decreased in both genotypes: *BrMYB38* (*BIT1*, Bra017218), *BrMYB51* (*HIG1*, Bra016553), *BrMYB73* (Bra010593), *BrDREB26* (Bra036022) and Integrase-type DNA-binding superfamily protein (Bra019087, Bra040309). Only one gene, *WRKY20* (Bra019095), showed decreased expression in Chiifu, but was not expressed in Kenshin. WRKY20 regulates ABA signaling and confers drought tolerance [56]. Since drought, salt and cold stresses are closely related to osmotic stress, the aforementioned genes might be involved in cold-responsiveness in *B. rapa*. The discrepancy of expression levels of *B. rapa* TF genes with those reported in previous studies with respect to abiotic stresses likely reflects variations in species-specific roles of TFs.

### RT-PCR validation of microarray analysis

To further confirm the microarray data and explain freezing tolerance in Chinese cabbage, we selected several categories of genes for RT-PCR analyses; specifically, genes induced in both genotypes, genes induced in Chiifu at the recovery stage, upstream and downstream genes of the CBF-regulon, and circadian rhythm-related genes (Figure S5A–F). A large number of genes were induced by cold-treatments in both genotypes (Figure S5A) and the recovery stage (Figure S5C), implying common genes for cold-response and restoration. Several genes that were specifically expressed in Chiifu (Figure S5B) would be required to confirm their role in cold-tolerance. Up-stream components and down-stream genes of *CBF* genes, core cold-responsive TFs in plants, showed similar patterns between Chiifu and Kenshin (Figure S5D, S5E), indicating that the CBF-regulon may not be the major cold-tolerant pathway or that a different regulatory mechanism was present in Chinese cabbage. Only one gene, *BrICEL-1*, was specifically expressed in Kenshin.

Circadian rhythm-associated genes expression is of particular interest. Specifically, PRR5 (PSUDO-RESPONSE REGULATOR 5) regulates expression of the timing of key TFs involved in clock-output pathways, including cold-stress [57]. PRR5 and PRR7 suppressed the expression of *CCA1*, *LHY* and *CBF* genes [58], which act as negative regulators for cold-responsive genes. However, expression of both negative and positive genes in *Arabidopsis* was induced by cold-treatment in both genotypes (Figure S5F), indicating different roles of these genes in cold-tolerance or the presence of different mechanisms for their tolerance in Chinese cabbage.

## Conclusion

A large number of genes were induced in both genotypes by cold-treatments, and expression of many genes including NHFs (Chinese cabbage specific genes) was differently regulated. In addition, many genes showed intrinsically different expression between Chiifu and Kenshin. All of these differently regulated genes are candidates for cold- or freezing-tolerance in Chinese cabbage. However, *Arabidopsis* and several other core regulatory genes of plants involved in cold-acclimation and -tolerance, such as circadian clock-associated genes, *CBFs* and CBF-targeted *COR* genes, showed similar patterns of expression in Chinese cabbage. For example, the freezing tolerance of Chinese cabbage shown in Figure 1 did not match previously known-gene expression. This discrepancy could have occurred for several reasons. Specifically, different classes of genes might respond to the same stimulus different in different plants. Timing of gene expression as found to be important to conferring freezing tolerance rather than expression levels. In Chinese cabbage, the function of NHF genes might be critical to freezing-tolerance. Since the conditions for cold-acclimation for Chinese cabbage will differ from those for *Arabidopsis*, 6 h at 4°C will not lead cold acclimation even though the expression of general cold responsive genes was induced. Intrinsically expressed levels of genes are important for cold-tolerance in plants. The fact that over-expression of cold-responsive genes (CBF-regulon and other genes) conferred freezing tolerance may imply that the tolerance is related to the levels of transcripts before cold-treatment or to requirement for the appropriate cold-acclimation conditions. Accordingly, further studies including transgenesis and genome-based sequence analyses of various genetic resources are necessary.

## Supporting Information

Figure S1 **Plant morphology of cold-treated Chinese cabbage.** Plant growth chamber was set to 16 h L/8 L D (Light period: 6∶00 a.m. –10∶00 p.m., Dark period: 10∶00 p.m. –6∶00 a.m.) photoperiod. C and K indicate Chiifu and Kenshin, respectively. Number 1 to 6 indicates the sample: 1 = control condition (22°C), 2 = 4°C treatment, 3 = 0°C treatment, 4 = −2°C treatment, 5 = −4°C treatment, 6 = 24 h recovery stage after all treatments.
(TIF)

Figure S2 **Transcriptome analyses of Chiifu and Kenshin shown by heat maps.** Heat maps of expression profiles of top-30 ranked induced genes upon −2°C treatment in Chiifu (A) and Kenshin (B) were compared. Absolute expression values are scaled by PI values. Detailed transcriptome information was described in Table S11 for Chiifu and Table S12 for Kenshin.
(TIF)

Figure S3 **Transcriptome analyses of Chiifu shown by heat maps.** Heat maps of expression profiles of over 4-fold up-regulated genes upon −2°C treatment in Chiifu were compared (see also Table S13). Absolute expression values are scaled by PI values.
(TIF)

Figure S4 **Transcriptome analyses of Chiifu and Kenshin shown by heat maps.** Heat maps of expression profiles of induced genes upon 4°C treatment in Chiifu or Keshin were also reconstructed from Addition file: Table S14. Absolute expression values are scaled by PI values.
(TIF)

Figure S5 **RT-PCR results of selected genes.** **A**, Genes induced by cold-treatments in both Chiifu and Kenshin. **B**, Genes specifically induced in Chiifu. **C**, Genes expressed at recovery stage in both genotypes. **D**, CBF-pathway up-stream genes. **E**, CBF-pathway down-stream genes. **F**, Circadian rhythm-related genes. NHF indicates a gene that was not found in the NCBI database.
(TIF)

Table S1 Primer sequences used in semi-qRT-PCR in order to confirm microarray analysis.
(XLSX)

Table S2 Primer sequences of stress-related genes (*SRG*) used in semi-qRT-PCR.
(XLSX)

Table S3 Summary of microarray data. PI (probe intensity) values were expressed by mean value of two replicated experiments. C and K indicate Chiifu and Kenshin, respectively. Number 1 to 6 indicates the sample: 1 = control condition (22°C), 2 = 4°C treatment, 3 = 0°C treatment, 4 = −2°C treatment, 5 = −4°C treatment, 6 = 24 h recovery stage after all treatments.
(XLSX)

Table S4 **Table S4A. U**p-regulated genes over 2-fold in Chiifu by cold-treatments. C and K indicate Chiifu and Kenshin, respectively. Number 1 to 6 indicates the sample: 1 = control condition (22°C), 2 = 4°C treatment, 3 = 0°C treatment, 4 = −2°C treatment, 5 = −4°C treatment, 6 = 24 h recovery stage after all treatments. **Table S4B.** Induced genes over 2-fold in Chiifu by cold-treatments. C and K indicate Chiifu and Kenshin, respectively. Number 1 to 6 indicates the sample: 1 = control condition (22°C), 2 = 4°C treatment, 3 = 0°C treatment, 4 = −2°C treatment, 5 = −4°C treatment, 6 = 24 h recovery stage after all treatments. **Table S4C. U**p-regulated genes over 2-fold in Kenshin by cold-treatments. C and K indicate Chiifu and Kenshin, respectively. Number 1 to 6 indicates the sample: 1 = control condition (22°C), 2 = 4°C treatment, 3 = 0°C treatment, 4 = −2°C treatment, 5 = −4°C treatment, 6 = 24 h recovery stage after all treatments. **Table S4D. Induced** genes over 2-fold in Kenshin by cold-treatments. C and K indicate Chiifu and Kenshin, respectively. Number 1 to 6 indicates the sample: 1 = control condition (22°C), 2 = 4°C treatment, 3 = 0°C treatment, 4 = −2°C treatment, 5 = −4°C treatment, 6 = 24 h recovery stage after all treatments. **Table S4E.** List of genes induced in both genotypes by cold-treatment. Genes having below 500 of PI values in all samples are eliminated. C and K indicate Chiifu and Kenshin, respectively. Number 1 to 6 indicates the sample: 1 = control condition (22°C), 2 = 4°C treatment, 3 = 0°C treatment, 4 = −2°C treatment, 5 = −4°C treatment, 6 = 24 h recovery stage after all treatments.
(XLSX)

Table S5 **Table S5A.** Down-regulated genes over 2-fold in Chiifu by cold-treatments. C and K indicate Chiifu and Kenshin, respectively. Number 1 to 6 indicates the sample: 1 = control condition (22°C), 2 = 4°C treatment, 3 = 0°C treatment, 4 = −2°C treatment, 5 = −4°C treatment, 6 = 24 h recovery stage after all treatments. **Table S5B.** Repressed- genes over 2-fold in Chiifu by cold-treatments. C and K indicate Chiifu and Kenshin, respectively. Number 1 to 6 indicates the sample: 1 = control condition (22°C), 2 = 4°C treatment, 3 = 0°C treatment, 4 = −2°C treatment, 5 = −4°C treatment, 6 = 24 h recovery stage after all treatments. **Table S5C.** Down-regulated genes over 2-fold in Kenshin by cold-treatments. C and K indicate Chiifu and Kenshin, respectively. Number 1 to 6 indicates the sample: 1 = control condition (22°C), 2 = 4°C treatment, 3 = 0°C treatment, 4 = −2°C treatment, 5 = −4°C treatment, 6 = 24 h recovery stage after all treatments. **Table S5D.** Repressed-genes over 2-fold in Kenshin by cold-treatments. C and K indicate Chiifu and Kenshin, respectively. Number 1 to 6 indicates the sample: 1 = control condition (22°C), 2 = 4°C treatment, 3 = 0°C treatment, 4 = −2°C treatment, 5 = −4°C treatment, 6 = 24 h recovery stage after all treatments. **Table S5E.** Down-regulated genes over 2-fold in both Chiifu and Kenshin by cold-treatments. C and K indicate Chiifu and Kenshin, respectively. Number 1 to 6 indicates the sample: 1 = control condition (22°C), 2 = 4°C treatment, 3 = 0°C treatment, 4 = −2°C treatment, 5 = −4°C treatment, 6 = 24 h recovery stage after all treatments. **Table S5F.** Repressed-genes over 2-fold in both Chiifu and Kenshin by cold-treatments. C and K indicate Chiifu and Kenshin, respectively. Number 1 to 6 indicates the sample: 1 = control condition (22°C), 2 = 4°C treatment, 3 = 0°C treatment, 4 = −2°C treatment, 5 = −4°C treatment, 6 = 24 h recovery stage after all treatments.
(XLSX)

Table S6 Functional classification of genotype-specific expressed genes/C and K indicate Chiifu and Kenshin, respectively. Number 1 to 6 indicates the sample: 1 = control condition (22°C), 2 = 4°C treatment, 3 = 0°C treatment, 4 = −2°C treatment, 5 = −4°C treatment, 6 = 24 h recovery stage after all treatments.
(XLSX)

Table S7 Induced genes by −4°C treatment in Chiifu. C and K indicate Chiifu and Kenshin, respectively. Number 1 to 6 indicates the sample: 1 = control condition (22°C), 2 = 4°C treatment, 3 = 0°C treatment, 4 = −2°C treatment, 5 = −4°C treatment, 6 = 24 h recovery stage after all treatments.
(XLSX)

Table S8 Induced genes by −4°C treatment in Kenshin. C and K indicate Chiifu and Kenshin, respectively. Number 1 to 6 indicates the sample: 1 = control condition (22°C), 2 = 4°C treatment, 3 = 0°C treatment, 4 = −2°C treatment, 5 = −4°C treatment, 6 = 24 h recovery stage after all treatments.
(XLSX)

Table S9 Induced genes by −2°C treatment in Chiifu. C and K indicate Chiifu and Kenshin, respectively. Number 1 to 6 indicates the sample: 1 = control condition (22°C), 2 = 4°C treatment, 3 = 0°C treatment, 4 = −2°C treatment, 5 = −4°C treatment, 6 = 24 h recovery stage after all treatments.
(XLSX)

Table S10 Induced genes by −2°C treatment in Kenshin. C and K indicate Chiifu and Kenshin, respectively. Number 1 to 6 indicates the sample: 1 = control condition (22°C), 2 = 4°C treatment, 3 = 0°C treatment, 4 = −2°C treatment, 5 = −4°C treatment, 6 = 24 h recovery stage after all treatments.
(XLSX)

Table S11 Genes up-regulated over 4-fold in Chiifu by −4°C treatment. C and K indicate Chiifu and Kenshin, respectively. Number 1 to 6 indicates the sample: 1 = control condition (22°C), 2 = 4°C treatment, 3 = 0°C treatment, 4 = −2°C treatment, 5 = −4°C treatment, 6 = 24 h recovery stage after all treatments.
(XLSX)

Table S12 Genes induced by Chilling (4°C) in both Chiifu and Kenshin. C and K indicate Chiifu and Kenshin, respectively. Number 1 to 6 indicates the sample: 1 = control condition (22°C), 2 = 4°C treatment, 3 = 0°C treatment, 4 = −2°C treatment, 5 = −4°C treatment, 6 = 24 h recovery stage after all treatments.
(XLSX)

Table S13 Cold-induced gene by either Chiifu or Kenshin. C and K indicate Chiifu and Kenshin, respectively. Number 1 to 6 indicates the sample: 1 = control condition (22°C), 2 = 4°C treatment, 3 = 0°C treatment, 4 = −2°C treatment, 5 = −4°C treatment, 6 = 24 h recovery stage after all treatments.
(XLSX)

Table S14 Clones that show significant expression values among no_hit_found (NTF). C and K indicate Chiifu and Kenshin, respectively. Number 1 to 6 indicates the sample: 1 = control condition (22°C), 2 = 4°C treatment, 3 = 0°C treatment, 4 = −2°C treatment, 5 = −4°C treatment, 6 = 24 h recovery stage after all treatments.
(XLSX)

Table S15 List of transcription factors on Br135K microarray and their expression levels. 22°C (C1, K1), 4°C (C2, K2), 0°C (C3, K3), −2°C (C4, K4), −4°C (C5, K5), 22°C (recovery stage, C6/K6). C and K represent Chiifu and Kenshin, respectively. Yellow shade indicates genes showing remarkable change in its expression.
(XLSX)
