# Supplementary figures and images for: Genome-Wide Transcriptome Analysis of Two Contrasting Brassica rapa Doubled Haploid Lines under Cold-Stresses Using Br135K Oligomeric Chip
Source: PLoS One. 2014 Aug 28;9(8):e106069. doi: 10.1371/journal.pone.0106069 (PMC4148347; doi:10.1371/journal.pone.0106069)

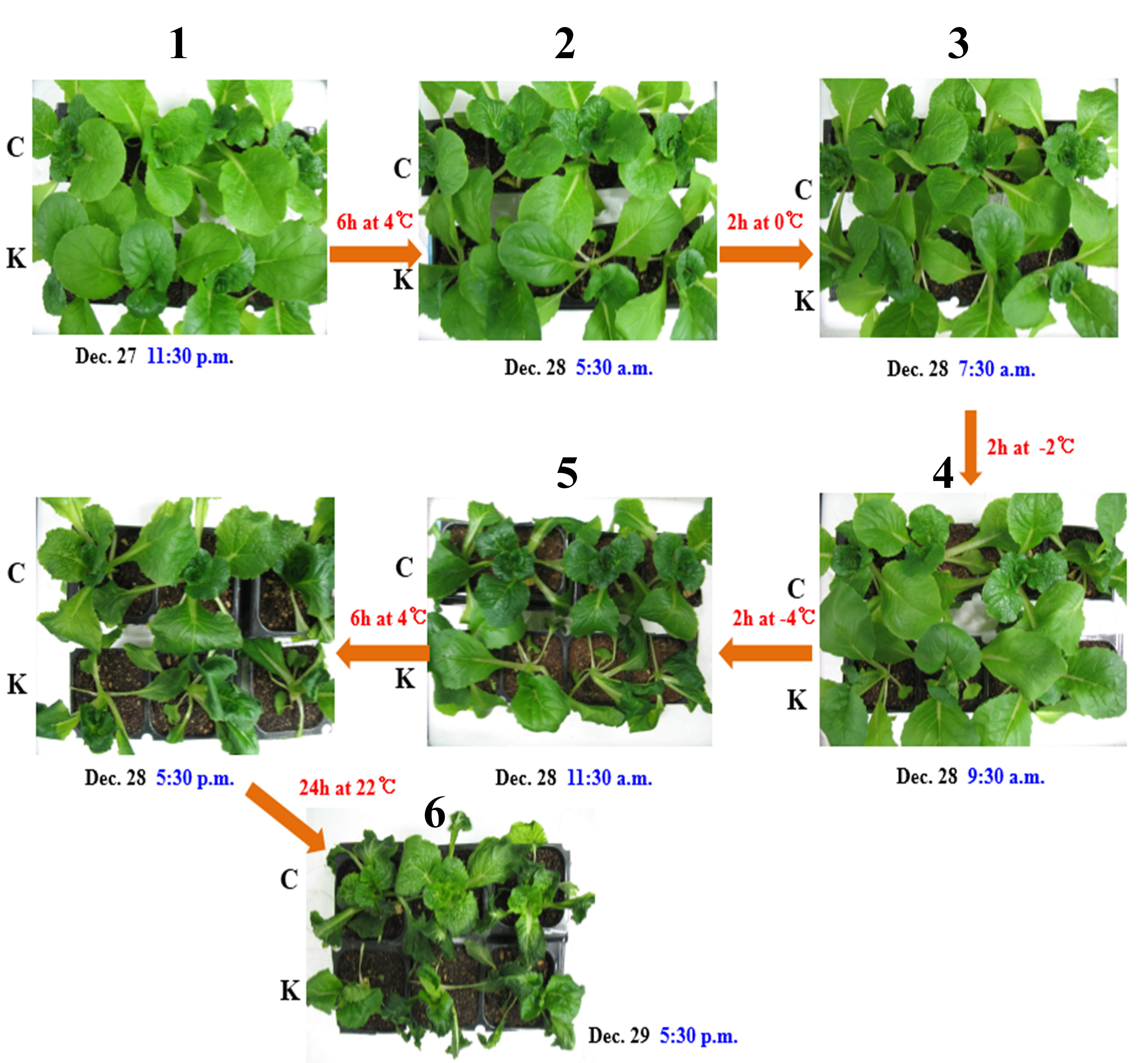

Supplement: Figure S1 — Plant morphology of cold-treated Chinese cabbage. Plant growth chamber was set to 16 h L/8 L D (Light period: 6∶00 a.m. –10∶00 p.m., Dark period: 10∶00 p.m. –6∶00 a.m.) photoperiod. C and K indicate Chiifu and Kenshin, respectively. Number 1 to 6 indicates the sample: 1 = control condition (22°C), 2 = 4°C treatment, 3 = 0°C treatment, 4 = −2°C treatment, 5 = −4°C treatment, 6 = 24 h recovery stage after all treatments. (TIF) [file pone.0106069.s001.tif]

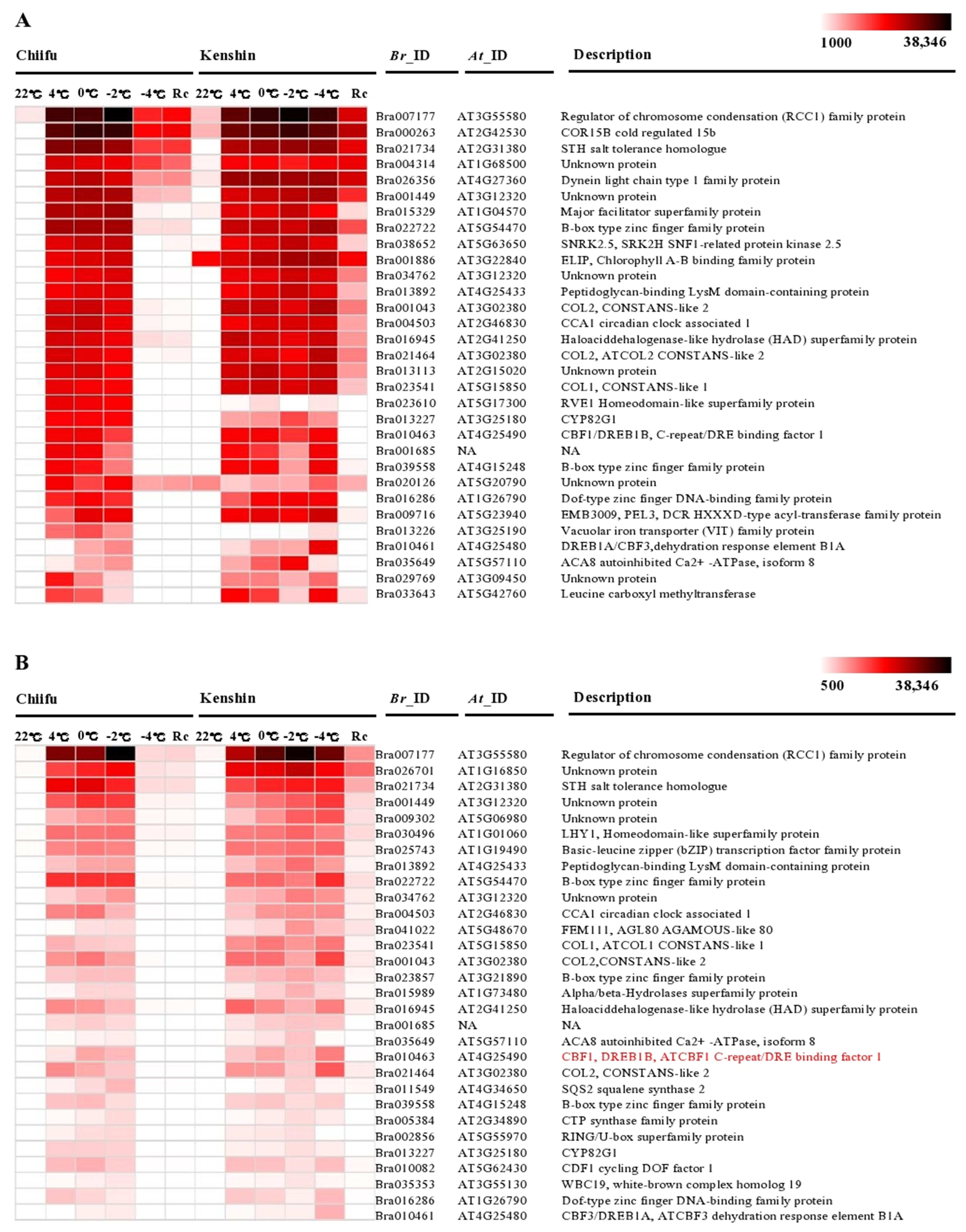

Supplement: Figure S2 — Transcriptome analyses of Chiifu and Kenshin shown by heat maps. Heat maps of expression profiles of top-30 ranked induced genes upon −2°C treatment in Chiifu (A) and Kenshin (B) were compared. Absolute expression values are scaled by PI values. Detailed transcriptome information was described in Table S11 for Chiifu and Table S12 for Kenshin. (TIF) [file pone.0106069.s002.tif]

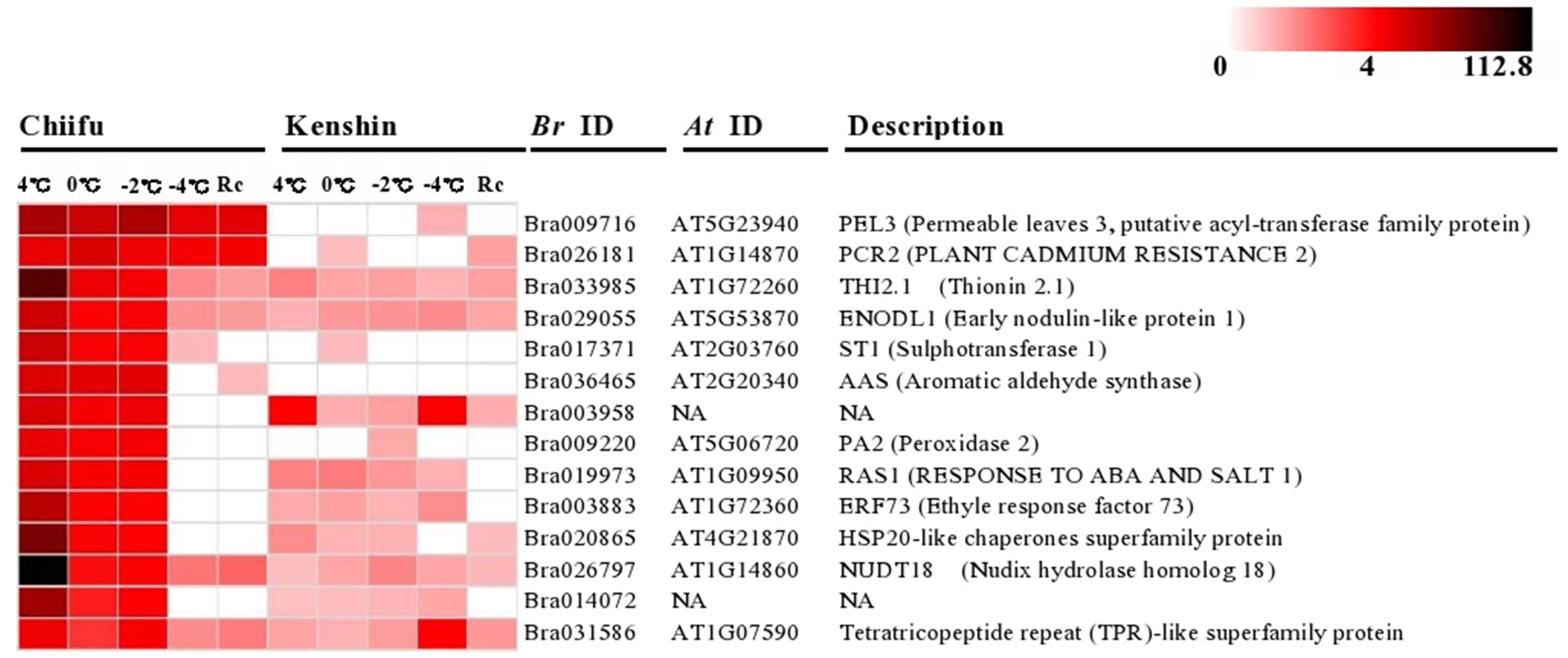

Supplement: Figure S3 — Transcriptome analyses of Chiifu shown by heat maps. Heat maps of expression profiles of over 4-fold up-regulated genes upon −2°C treatment in Chiifu were compared (see also Table S13). Absolute expression values are scaled by PI values. (TIF) [file pone.0106069.s003.tif]

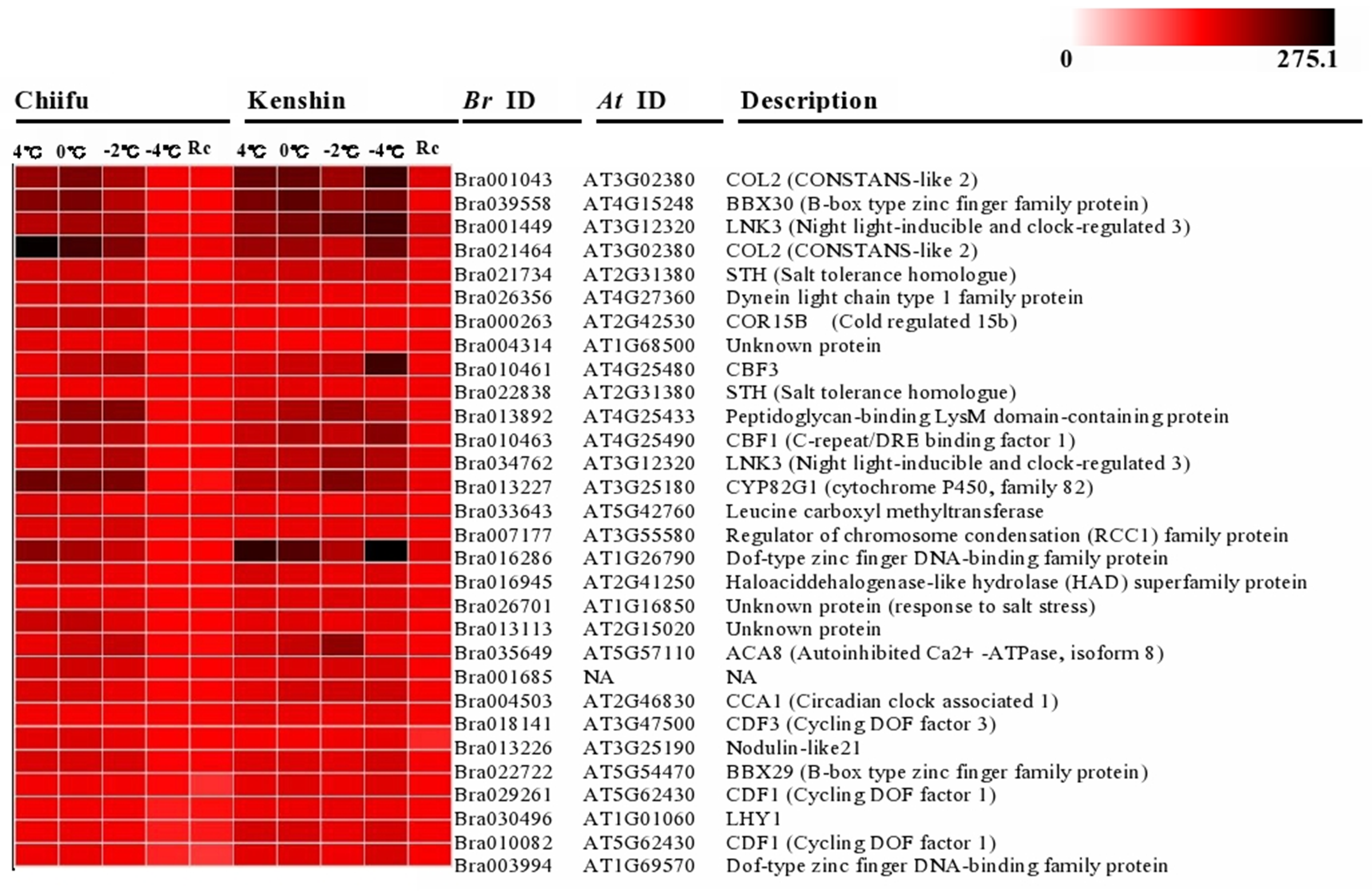

Supplement: Figure S4 — Transcriptome analyses of Chiifu and Kenshin shown by heat maps. Heat maps of expression profiles of induced genes upon 4°C treatment in Chiifu or Keshin were also reconstructed from Addition file: Table S14. Absolute expression values are scaled by PI values. (TIF) [file pone.0106069.s004.tif]

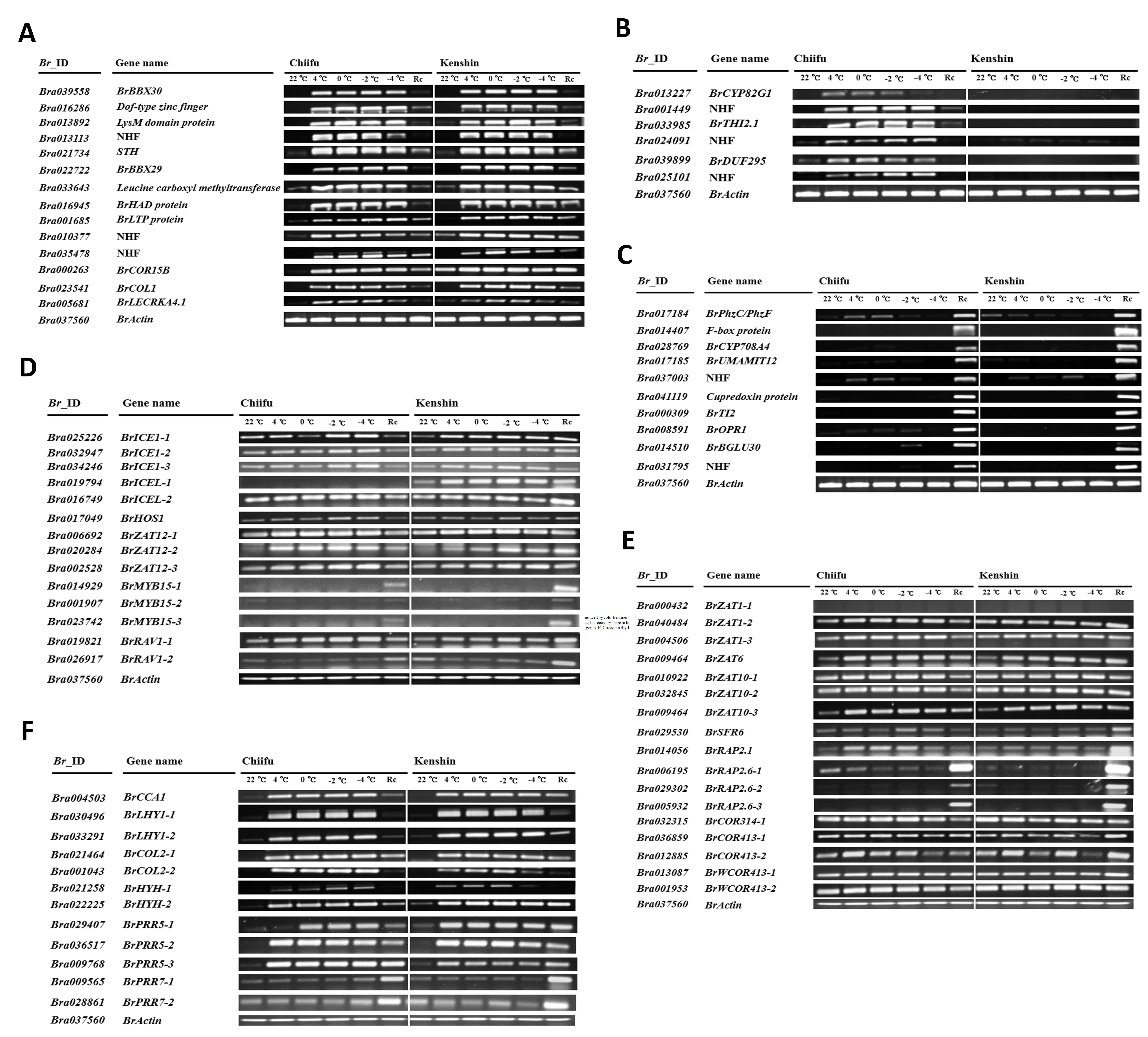

Supplement: Figure S5 — RT-PCR results of selected genes. A, Genes induced by cold-treatments in both Chiifu and Kenshin. B, Genes specifically induced in Chiifu. C, Genes expressed at recovery stage in both genotypes. D, CBF-pathway up-stream genes. E, CBF-pathway down-stream genes. F, Circadian rhythm-related genes. NHF indicates a gene that was not found in the NCBI database. (TIF) [file pone.0106069.s005.tif]
